# A Clinical Medication Review Focused on Deprescribing in Older Patients With Hyperpolypharmacy: A Mixed‐Methods Feasibility Study

**DOI:** 10.1111/bcpt.70184

**Published:** 2025-12-28

**Authors:** Gert Baas, Mette Heringa, Sanne Verdoorn, Henk‐Frans Kwint, Eman Badawy, Jacobijn Gussekloo, Marcel Bouvy

**Affiliations:** ^1^ SIR Institute for Pharmacy Practice and Policy Leiden the Netherlands; ^2^ Department of Pharmacoepidemiology and Clinical Pharmacology University of Utrecht Utrecht the Netherlands; ^3^ Center for Medicine for Older People Leiden University Medical Center Leiden the Netherlands

**Keywords:** clinical medication review, deprescribing, feasibility, older patients, polypharmacy

## Abstract

To address challenges in deprescribing, we investigated the feasibility of an intervention consisting of a clinical medication review (CMR) focused on deprescribing, supported by a training programme for healthcare providers (HCPs) among older patients with hyperpolypharmacy (≥ 10 chronic medications) in primary care. A mixed‐methods feasibility study was conducted in six Dutch community pharmacies using Bowen's framework. The intervention comprised HCP training and a five‐step deprescribing‐focused CMR. Within 6 Bowen domains, 18 outcomes were assessed through (patient) questionnaires, interviews (patients, HCPs), process parameters, and medication dispensing data. Five pharmacists conducted CMRs with 24 patients (median age: 84.5 years). The intervention was well accepted by patients and HCPs. However, barriers emerged regarding *implementation* and *practicality*. Consultations lacked complete discussion of patient concerns, and pharmacists reported varying levels of confidence in making deprescribing decisions. Time constraints limited the incorporation of deprescribing into CMRs. On average, 1.3 medications per patient were deprescribed. Within a setting of motivated and CMR‐experienced HCPs, adding a focus on deprescribing to CMRs for older patients with hyperpolypharmacy was feasible and well received. Feasibility was supported by high acceptability and deprescribing potential, though barriers in implementation and practicality indicate the need for further evaluation in broader primary care settings.

## Plain Language Summary

Many older people take many medicines at once, which can cause side effects or unnecessary treatments. In this study, pharmacists and doctors worked together to review patients' medicines and decide which could be safely reduced or stopped. After training, pharmacists carried out medication reviews with 24 older patients who used at least 10 medicines. The approach was well accepted and led to fewer medicines being used safely. Some practical challenges, such as limited time and confidence in stopping medicines, still need to be addressed before this method can be applied more widely.

## 1 Introduction and Background

As society ages, the prevalence of polypharmacy among older patients increases. For example, 45% of patients aged 75 years and older use five or more chronic medications in the Netherlands [1]. While polypharmacy is often necessary to manage multimorbidity, it also introduces significant risks, including an increased likelihood of drug‐related problems (DRPs). A common problem is the use of potentially inappropriate medications (PIMs) in older patients: drugs for which the potential harm outweighs the benefits or that lack efficacy in this population [2, 3, 4] Hyperpolypharmacy, defined as the use of 10 or more chronic medications [5], further amplifies these risks and significantly increases the likelihood of DRPs [6].

To address the negative effects of (hyper)polypharmacy, there is growing interest in deprescribing. Deprescribing involves reducing or stopping medications under the supervision of healthcare providers (HCPs), aiming to minimize adverse drug reactions and improve health outcomes [7, 8]. Evidence suggests that deprescribing can be particularly beneficial for older patients with hyperpolypharmacy, frailty, cognitive impairment, or those using high‐risk medications such as benzodiazepines, anticholinergic drugs, and opioids [9, 10, 11]. However, the effectiveness of deprescribing interventions varies, influenced by factors such as patient characteristics, attitudes of HCPs, and structural barriers or facilitators in clinical practice. While some studies report positive outcomes, including reduced medication burden, other studies highlight challenges in implementation [12, 13, 14].

In previous research, key barriers to deprescribing in primary care have been shown to be related to knowledge, skills, and organizational factors [12, 14, 15], including the lack of education and comprehensive guidelines. In 2020, this last barrier was overcome in the Netherlands by the introduction of a multidisciplinary deprescribing guideline, including 10 drug‐specific deprescribing fact sheets on how and when to deprescribe in (frail) older patients [16]. However, only the availability of a guideline is not sufficient on its own to realize its implementation [17]. For effective implementation of deprescribing, improvements in patient selection and collaboration among HCPs are important. To strengthen skills, targeted education and training for HCPs is required [12, 13]. To address these organizational challenges, integration of deprescribing into a clinical medication review (CMR) is proposed. Key elements that make CMRs suitable for implementation of deprescribing, including the involvement of both the general practitioner (GP) and the community pharmacist (CP), along with a patient consultation ensuring a patient‐centred approach that facilitates shared decision‐making. It has been shown that CMRs led to more changes in medication, a reduction in the number of DRPs and reduction of number of prescribed drugs in use [6, 18, 19, 20, 21]. In addition, Dutch research showed that the number of health‐related complaints can be reduced and quality of life (EQ‐VAS) can be improved [20]. In the Netherlands, CMRs are routinely conducted by CPs and GPs.

To address current challenges in deprescribing, we designed an intervention consisting of a CMR focused on deprescribing in older patients with hyperpolypharmacy, along with a training programme and toolbox to support HCPs in effectively implementing the Dutch deprescribing guideline in primary care [16]. This study aims to test the feasibility of this intervention

## 2 Materials and Methods

### 2.1 Study Setting and Design

The study was conducted in accordance with the Basic & Clinical Pharmacology & Toxicology policy for experimental and clinical studies [22]. This feasibility study was conducted in a Dutch primary care setting. Dutch CPs and GPs regularly collaborate in a five‐step CMR process for older patients with polypharmacy, following the Dutch multidisciplinary CMR guideline [23]. The study was performed in six community pharmacies that planned to conduct five CMRs focused on deprescribing each.

Bowen's Framework [24] was utilized to evaluate the feasibility using a mixed‐methods approach. This framework comprises eight domains that help determine whether an intervention is appropriate and ready for further testing. Eighteen outcomes in six domains were defined, taking into account the perspectives of patients, GPs, and CPs based on nine data sources (see Table 1).

**TABLE 1:** Feasibility domains and outcomes from perspectives of patients, general practitioners (GPs), and community pharmacists (CPs).

Domain^a^ (Bowen [24])	Outcomes		Perspective	Data source	#
1. Acceptability	1.1	Percentage of patients who rate their likelihood of recommending the intervention to others as 8 or higher (scale from 0 to 10)	Patients	Patient questionnaire	*n* = 23
	1.2	Opportunities and challenges regarding the deprescribing intervention		Patient interviews	*n* = 5
	1.3	Percentage of pharmacists who, after training, feel capable of implementing the intervention	CPs	Training evaluation questionnaire	*n* = 6
	1.4	Perceived appropriateness regarding the deprescribing intervention elements	CPs	Interviews with HCPs	*n* = 6
			GPs		*n* = 4
2. Demand	2.1	Extent to which patients were willing to participate, and reasons for (not) participating the study/intervention	Patients	Recruitment lists	*n* = 6
				Patient interviews	*n* = 5
	2.2	Reasons why HCPs were willing to conduct study/intervention	CPs	Interviews with HCPs	*n* = 6
			GPs		*n* = 4
3. Implementation	3.1	Ratio of discussed vs. non‐discussed topics during patient consultation and their relative importance	Patients	Patient questionnaire	*n* = 23
	3.2	Factors affecting HCPs implementing (deprescribing) actions in the context of the intervention	CPs	Interviews with HCPs	*n* = 6
			GPs		*n* = 4
4. Practicality	4.1	Essential prerequisites to conduct the intervention (CMR) in relation to the organization of daily practice	CPs	Interviews with HCPs	*n* = 6
			GPs		*n* = 4
	4.2	Time required for the intervention	CPs	CMR process data	*n* = 24
	4.3	Distribution of consultation locations			
5. Adaptation	5.1	Perceived value of the intervention on the targeted population and conduct of a deprescribing focused CMR	CPs	Interviews with HCPs	*n* = 6
			GPs		*n* = 4
6. Limited testing of efficacy	6.1	Number of medications^b^ stopped and/or dose reduced after 3 months	Patients	Dispensing data	*n* = 20^c^
	6.2	Percentage patients with at least one deprescribing recommendation	Patients	CMR data	*n* = 24
	6.3	Percentage patients with at least one implemented deprescribing action			
	6.4	Number and type of DRPs focused on deprescribing			
	6.5	Implementation rates (deprescribing) recommendations			
	6.6	Frequency and severity of health‐related complaints with impact on daily life (t = 0 vs. t = 3 months)	Patients	Questionnaire	t = 0; *n* = 24 t = 3; *n* = 21

^a^ Six of the eight domains of Bowen’s framework are used and presented in Table 1. The domain of ‘integration’ was excluded because CMR is already an established intervention in the Netherlands. The ‘expansion’ domain was excluded as this study focuses on a narrower subset of patients already participating in regular CMRs.

^b^ Includes only medications dispensed within the MDD system, as these were assumed to be used chronically

^c^ Data analysed for 20 of 24 included patients: 2 had no data, 1 did not use the MDD system, and 1 had incomplete data due to death.

### 2.2 Intervention on HCP Level and Patient Level

The intervention consisted of a CMR focused on deprescribing (patient level) preceded by training of the participating HCP (HCP level).

A full‐day training programme for CPs was developed based on previous training programmes [12], covering the main barriers identified in previous research and the topics covered in the guideline: (1) the organization of CMR focusing on deprescribing; (2) communication skills to support deprescribing consultations; and (3) the application of a toolbox in practice. This toolbox consisted of (i) drug‐specific deprescribing fact sheets; (ii) a consultation aid leaflet with recommendations for deprescribing consultations; (iii) materials to inform GPs including an information letter and a presentation to be used in a local pharmacotherapy audit meeting (PTAM), covering the same aspects as in the pharmacist's training. PTAMs are structured interprofessional meetings between CPs and GPs in the Netherlands to discuss pharmacotherapy and optimize prescribing on selected themes [25].

**FIGURE 1:**
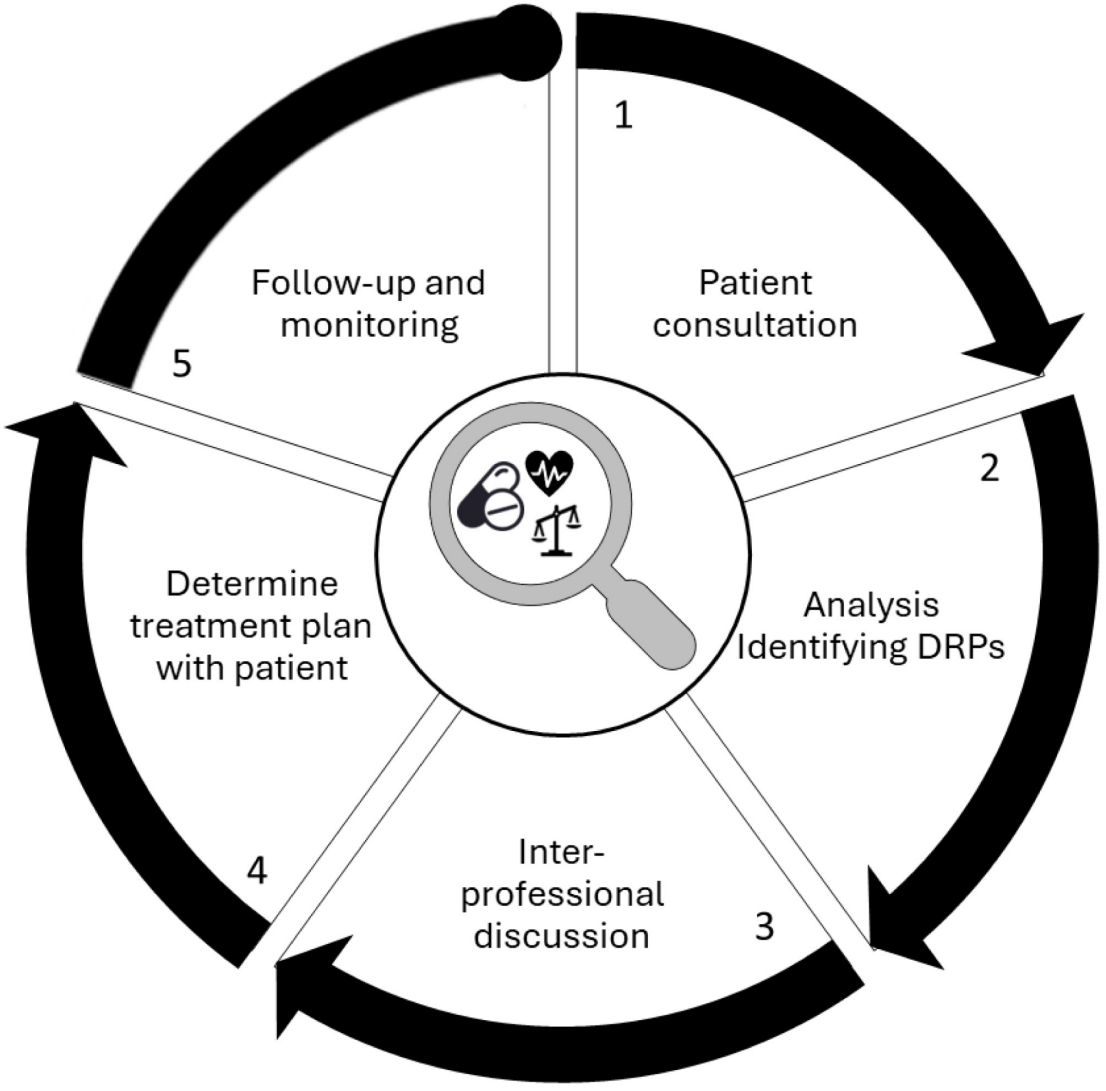
Five step approach of a CMR according to the Dutch multidisciplinary guideline ‘Polypharmacy in the Elderly’ [23] with special focus on deprescribing in our study. The CMR starts with a consultation (step 1) between the patient and the CP, using input from the questionnaires to understand the patient’s preferences and health‐related issues. This includes identifying any medications the patient prefers to continue or discontinue and any adverse effects they may be experiencing. This is followed by a pharmacotherapeutic analysis by the CP to identify potential DRPs and opportunities for deprescribing (step 2). The CP and GP then discuss the identified DRPs, patient preferences and possible actions, including deprescribing (step 3). If needed, the CP or GP consults other HCPs. Together with the patient, the CP prioritizes the actions to be taken, resulting in a pharmaceutical care plan (step 4). All deprescribing actions are monitored through follow‐up by either the CP, nurse practitioners, or GPs, with interprofessional communication as needed, to adjust tapering based on the patient’s health or withdrawal symptoms (step 5).

The patient‐level intervention is a five‐step CMR, in accordance with the Dutch multidisciplinary guideline, but with a special focus on deprescribing (Figure 1) [23]. Before the patient consultation (step 1), patients completed questionnaires on health‐related problems and preferences [26]. The questionnaire served two specific purposes: (i) to measure the patient's health status and enable monitoring of changes over time following the intervention, and (ii) to provide the pharmacist with preliminary insight into the patient's health problems and preferences, thereby facilitating a more focused consultation and, where appropriate, linking these aspects to deprescribing decisions.

### 2.3 Study Participants

#### 2.3.1 HCP Selection and Inclusion

CPs in the region of Leiden (the Netherlands) were recruited through a regional pharmacists' network. The aim was to recruit a minimum of five CPs with collaborating GPs to test feasibility in different settings.

#### 2.3.2 Patient Selection and Inclusion

Patients aged 75 years or older who used a multidose drug dispensing (MDD) system with hyperpolypharmacy (≥ 10 drugs) were eligible. In the Netherlands, approximately 562 000 people (about 3% of the population) receive their medicines in an MDD system, the majority being older patients with polypharmacy; in 2023, around 20% of those aged 75 years and older used an MDD [27]. For this study, patients with MDD systems were selected for three reasons. First, MDD use can serve as a practical marker for identifying older patients at increased risk of medication‐related problems and frailty [28]. Second, MDD systems enable close monitoring of medication use through dispensing data. And third, the MDD system can be used as a practical tool to support deprescribing by facilitating the implementation of tapering schedules and thereby reducing the burden on older patients.

Patients were excluded in the case of (1) a life expectancy of ≤ 6 months, (2) cognitive impairment, (3) residence in nursing homes, (4) a CMR conducted within the past 12 months, and (5) patients for whom the GP was not the primary prescriber.

CPs generated a list from their pharmacy information system with patients aged 75 years and over with polypharmacy and a MDD. They screened the list collaboratively with GPs for exclusion criteria, particularly for exclusion criteria related to limited life expectancy and cognitive impairment. Eligible patients were then non‐systematically invited by telephone, with CPs being instructed to do so without applying any fixed order (e.g., alphabetical or first‐listed) or reviewing additional patient information. Interested patients received information packages, including informed consent forms. Recruitment continued until at least five patients per pharmacy agreed to participate.

### 2.4 Data Collection and Analysis

Data were collected from nine data sources between August 2021 and March 2022.

#### 2.4.1 Patient Level Data Collection

All forms and letters were pretested by older patients recruited through Zorgbelang Inclusief's patient panel and language ambassadors (trained laypersons specialized in assessing readability of health communication materials). The pretest focused on clarity and readability for older patients:

1 Health‐related complaints and preferences
  Patients completed questionnaires addressing health‐related complaints and preferences at t = 0 and t = 3 months [26]. The research team conducted these questionnaires by telephone and registered responses in CASTOR EDC, an online data collection platform for clinical research.
  Twelve health‐related complaints were scored using a Visual Analogue Scale (VAS; range 0–10) for severity and a 5‐point Likert scale to evaluate their impact on daily life. Data were categorized per patient into ‘complaints with impact’ on daily life. A ‘complaint with impact’ on daily life was defined as one with a severity score of ≥ 5 for the VAS and moderate, severe, or extreme influence on daily life (i.e., ≥ 3 points on the 5‐point Likert scale) [20].
2 Patient questionnaire
  A patient questionnaire based on the validated patient‐reported experience measurement (PREM) version for chronic health [29] and adjusted to reflect the topics of the deprescribing guideline [16] was conducted at t = 1 month (Data S1). It measured overall satisfaction with the CMR, including six items on specific aspects of the CP's service (5‐point Likert scale). Implementation of the deprescribing consultation as trained was evaluated by analysing the proportion of questionnaire‐derived topics discussed during patient consultations and their perceived importance, assessed through nine items on preferred consultation content (5‐point Likert scale). Quantitative data were analysed descriptively, and free‐text responses were thematically classified by two researchers (G.B. and M.H.).
3 Recruitment lists
  Pharmacists recorded reasons for exclusion and patient refusals. The percentage of eligible patients and their willingness to participate were analysed. Reasons for non‐participation were categorized.
4 Dispensing data
  Dispensing data from the pharmacy information system were collected for the period from 1 April 2020 to 31 March 2022 (24 months). Data included ATC code, dispensing date, medication name, amount, and dosing schedule of the medication. Number and type of medications in use at t = 0 and t = 3 months were compared independently by two researchers (E.B. and S.B.). Deprescribing was defined as stopping or reducing medication persistently after 3 months using a stepwise approach. In the analysis, medication reduction included both dose reductions and substitutions with a less potent alternative. At ATC‐5 level, medications were checked for continuation. If present at t = 3 months, the daily dose was assessed to identify dose reductions. If not present, substitutions within the same therapeutic group (ATC‐2 level) were identified. In such cases, the research team evaluated equivalence and potency according to guidelines, documenting lower potency as ‘dose lowered’. If no substitution was identified, the medication was documented as ‘stopped’. As MDD‐packaged medications were assumed to be used chronically, deprescribing was assessed for medication within this system.
5 Patient interviews
  After completion of the intervention, semi‐structured interviews were conducted with patients to determine their experiences and the feasibility of the intervention. Researchers used a pragmatic purposive approach inviting patients for interviews, aiming to recruit one patient per pharmacy and ensuring equal distribution across the pharmacies.
  The topic guide was developed based on previously conducted research on deprescribing [30, 31, 32, 33] and focused on the deprescribing process, the information supply on pros and cons, and shared decision making (Data S2, table A). Patients were interviewed by G.B. or A.A. by telephone, and interviews were audio recorded. Content analysis was performed by two independent researchers (G.B. and E.B.) in NVivo (version R14.23.2). Researchers independently summarized key content into codes, which were directly categorized within Bowen's framework. Discrepancies were discussed until consensus was reached.
6 CMR data
  CPs documented identified DRPs based on the Hepler and Strand's classification system, recommendations, and implemented recommendations [34]. Two researchers (G.B. and E.B.) independently checked the completeness and consistency of documented data. Differences were resolved by consulting a third researcher (either S.V. or M.H.). Recommendations were classified as related to deprescribing if they involved stopping medication, reducing its dosage, or substituting it with a less potent alternative.

#### 2.4.2 Data Collection at Level of HCPs

7 Training evaluation questionnaire
  CPs completed a paper questionnaire at the end of the training (Data S3). The questionnaire assessed self‐experienced deprescribing competencies in three key areas: organization, communication, and deprescribing skills, using a total of 8 items rated on a 5‐point Likert scale. Additionally, participants responded to three open‐ended questions regarding the most and least valuable aspects of the training, as well as suggestions for improvement. CPs also responded to a general statement regarding whether the training met their expectations. The percentage of pharmacists who scored at least 4 out of 5 on the Likert scale for each of the key areas was calculated. Free text information was thematically classified by two researchers (G.B. and M.H.).
8 Interviews with HCPs.
  Semi‐structured interviews were conducted with participating HCPs to determine their experiences, satisfaction, and the feasibility of the intervention. All participating CPs were interviewed, and each CP suggested a collaborating GP to be interviewed.
  Topic guides were developed based on previously conducted research on deprescribing and signals identified during the monitoring of the study [12, 14, 15].
  The CP topic guide covered their experiences with the provided training and tools and in conducting CMRs focused on deprescribing (Data S2, table B). The GP topic guide addressed their views on the deprescribing intervention and their overall assessment of its feasibility and effectiveness (Data S2, table C).
  Online interviews were conducted through Microsoft Teams, recorded and transcribed verbatim. Content analysis was performed by two independent researchers (G.B. and E.B.) in NVivo (version R14.23.2) [35]. Researchers independently summarized key content into codes, which were directly categorized within Bowen's framework [24] and themes within domains were identified. Discrepancies were discussed until consensus was reached.
9 CMR process data
  Location of patient consultation and time spent on the different CMR steps were documented by CPs in CASTOR EDC.

### 2.5 Ethics and Confidentiality

The Medical Research Ethics Committee (MREC) NedMec determined that the study proposal (21/672) was not subject to the Medical Research Involving Human Subjects Act (WMO). All participants gave written informed consent. To ensure patient privacy, all data were anonymized. Data collection and management were conducted using CASTOR EDC, a secure online data management platform

## 3 Results

Six CPs (five female, one male) followed the training programme. Five of them together included 24 patients (2–8 patients per CP). Feasibility results are presented according to the six relevant domains of Bowen's Framework.

### 3.1 Acceptability

Among the 23 patients who completed the questionnaire, the median satisfaction score, measured by the likelihood of recommending the intervention, was 8 out of 10 (IQR: 7–9) (outcome 1.1). In interviews, patients expressed overall positive reactions to the deprescribing intervention (outcome 1.2). Trust in the process and the collaborative approach with other HCPs were recurring themes [Table 2, PA‐Q1]. Patients viewed the intervention as safe and effective, with no concerns about potential harm [Table 2, PA‐Q2].

**TABLE 2:** Quotes from the interviews.

Domains [Bowen [24]]	Perspective	Quote number	Quote
Acceptability	**Opportunities and challenges regarding the deprescribing intervention (outcome 1.2)**		
	Patient	PA‐Q1	‘“Okay, I'll give it a try.” Then she told me she would discuss it with the GP and that I would be called back afterwards. Nothing happens without my and the GP's involvement. I really appreciated that, as it reassured me that they would not just stop a medication without careful consideration.’
		PA‐Q2	‘I was asked to participate, and I thought, well, it cannot hurt, right?’
	**Perceived appropriateness regarding the deprescribing intervention elements (outcome 1.4)**		
	GP	GP‐Q1	‘That remains, of course, quite exciting because, for example, interpreting certain clinical information of a patient raises the question of whether this should be left to the pharmacist.’
	CP	CP‐Q4	‘Now, I can provide a solid rationale to the doctor. I send them that piece of supporting evidence. It makes things easier for me. And that's what I did.’
		CP‐Q5	‘I found it helpful to discuss communication during the training, especially when a doctor disagrees or when a patient finds it difficult to taper off—how do you approach that?’
		CP‐Q6	‘I made extensive use of the deprescribing fact sheets, which I had not really looked at before, so it was after the training day that I started using them much more.’
Demand	**Extent to which patients were willing to participate, and reasons for (not) participating the study/intervention (outcome 2.1)**		
	Patient	PA‐Q7	‘Well, I think it's useful because I believe I can use fewer medications, and that's why I'm participating.’
		PA‐Q8	‘Because I wanted to know if all the medications I'm taking are really necessary.’
	**Reasons why health care professionals were willing to conduct study/intervention (outcome 2.2)**		
	CP	CP‐Q9	‘MDD system users who have been on the same medication for two or three years are important because they are often not closely monitored by the GP, who assumes everything is fine and just continues to approve the prescriptions. These are precisely the people you want to focus on, as they likely no longer need certain medications.’
	GP	GP‐Q10	‘I think this is the target group where the most gains can be made most easily. These are people who are often well‐monitored, use a lot of medication, and therefore have a higher chance that there is something among them that should be stopped.’
Implementation	**Factors affecting HCPs implementing (deprescribing) actions in the context of the intervention (outcome 3.2)**		
	CP	Theme: patient motivation	
		CP‐Q11	‘I always ask; “What would you think if we stopped this?”. Sometimes the response is, “As far as I'm concerned, you can stop everything; I want as few pills as possible,” and sometimes people find it a bit daunting, “Is that even possible?” especially if a cardiologist initially prescribed it. I always try to follow up by asking where the fear comes from and what the problem is.’
		CP‐Q12	‘You call someone and say, “We would like to see if you could use fewer medications.” And then you notice that people are generally enthusiastic about it. My patients were all very positive, saying things like, “Yes, that's great, please come by.”’
		Theme: decision‐making	
		CP‐Q13	‘Well, he said, “Yes, things are going well, and I'm doing this,” and he was also in his 80s, still working and taking care of his wife. It was very difficult to decide, and it was also hard for me to say which medications could be stopped.’
		CP‐Q14	‘I sometimes find it challenging because you do not want to stop all medications at once. Especially with vulnerable elderly patients. If something goes wrong, it's hard to pinpoint the cause. Deciding where to start or what to stop simultaneously can be difficult.’
		Theme: fear of negative outcomes and medication classes	
		CP‐Q15	‘I think that both the GPs and I are afraid that something might happen, and that it would be directly due to stopping the blood thinners.’
		CP‐Q16	‘Well, especially regarding antipsychotics, it depends on the indication. If they are used for sleep medication, it is easier to taper off, but if someone has bipolar disorder or a serious psychiatric condition, then it is, of course, more difficult to stop such medications.’
		Theme: interprofessional collaboration	
			
	GP	GP‐Q17	‘We, as general practitioners, are being asked to work in the specialist's field, and I wonder if that should be our role.’
		GP‐Q18	‘That's the difference—when working with a pharmacist, versus doing it alone, you are less inclined to take a conservative approach of “just leave it,” as any change can also lead to complications.’
		Theme: patient understanding	

GP‐Q19	‘Hindering factors include that people often do not fully understand why they should stop taking a particular medication.’
Practicality	**Essential prerequisites to conduct the intervention (CMR) in relation to the organization of daily practice (outcome 4.1)**		
	CP	CP‐Q20	‘Seeing five patients a week, if you still have to process everything—aside from this research—I find that far too much combined with all the daily tasks. So, one patient a week is more than enough.’
		CP‐Q21	‘But I do find it annoying that something like this takes so long. I asked the GP about it at least 20 times, then the practice nurse, and then the assistant. The assistant eventually took action, but did not inform me.’
	GP	GP‐Q22	‘I have to say that the pharmacist consultations I had, really felt like piecing together a puzzle for each individual. It took quite a bit of time’
		GP‐Q23	‘Yes, I appreciate that because it means that if we have more trust in the pharmacist, over time, perhaps more can be handled by them, and they might be able to take some tasks off our hands.’
Adaptation	**Perspectives on the impact of the intervention on the targeted population and conduct of a deprescribing focused CMR (outcome 5.1)**		
	GP	GP‐Q24	‘I believe deprescribing should always be part of a clinical medication review.’
	CP	CP‐Q25	‘This is a good way to start the conversation, and eventually, everything naturally comes up. I also felt the conversation went a bit faster because you are more focused on finding the right medication. The patient also knows, “Well, that's what we are going to do.”’

The training was positively received by CPs, who highlighted its practical relevance. While all CPs felt capable of performing CMRs focused on deprescribing in collaboration with other HCPs, confidence in applying deprescribing knowledge varied, with 67% feeling capable of using deprescribing fact sheets in practice (outcome 1.3). They valued practical case studies and consultation exercises the most. The interviews confirmed that CPs considered the training valuable, especially with regard to enhancing consultation skills [Table 2, CP‐Q5] and knowledge on deprescribing fact sheets [Table 2, CP‐Q6].

The interviews also showed that CPs and GPs generally found the intervention appropriate, especially as it integrates deprescribing into the existing CMR process (outcome 1.4). GPs emphasized the importance of a collaborative approach, expressing concerns that CPs might interpret clinical values differently when making deprescribing decisions [Table 2, GP‐Q3]. CPs found the toolbox, such as fact sheets, useful for facilitating discussions with GPs [Table 2, CP‐Q4].

### 3.2 Demand

**FIGURE 2:**
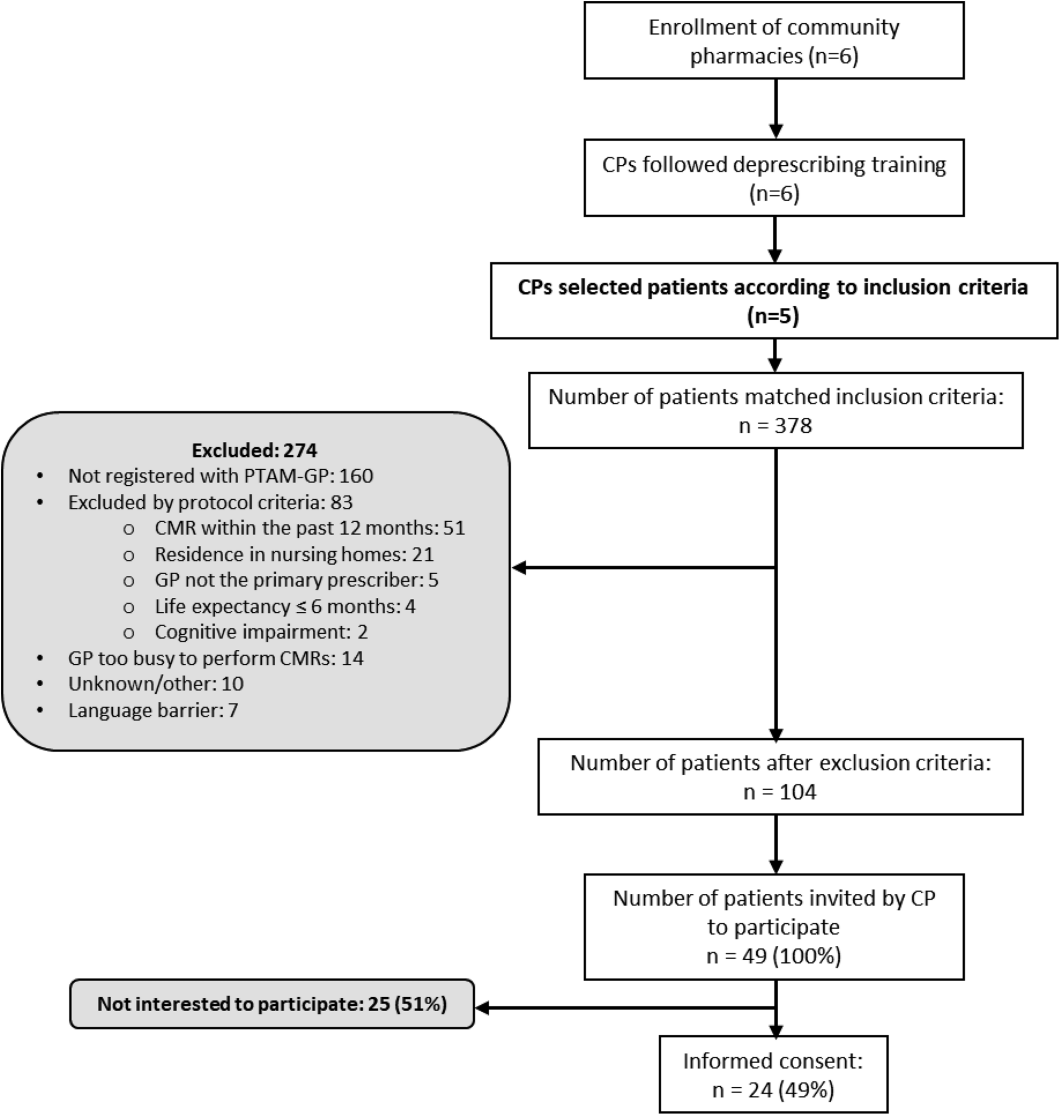
Flow‐chart of patient recruitment.

In total, 378 patients matched the inclusion criteria. After applying the exclusion criteria, 104 patients (28%) remained eligible (Figure 2). Of those contacted, 49% (*n* = 24) agreed to participate in the study (outcome 2.1) with an average age of 84.5 years and 63% being female. Main reasons for not participating were lack of time and/or interest, or feeling overburdened due to other appointments with physicians. From interviews, patients who did participate expressed curiosity and a desire to reduce their medication as key motivators [Table 2, PA‐Q7]. Patients valued the opportunity to reassess their medication use [Table 2, PA‐Q8].

With regard to HCPs, motivations of GPs and CPs were different (outcome 2.2). CPs saw the intervention as an opportunity to address deprescribing in patients using MDD systems, who are often overlooked [Table 2, CP‐Q9]. GPs viewed the intervention as an opportunity to address overprescribing in high‐risk older patients, despite noting that this group is generally well monitored [Table 2, GP‐Q10]. Both groups recognized the need for more structured approaches to managing medication use in older patients.

### 3.3 Implementation

Implementation was evaluated based on topics discussed during consultations according to patients (outcome 3.1) and challenges faced by HCPs (outcome 3.2).

Based on the patient questionnaires (*n* = 23), most frequently discussed topics included the (dis)advantages of deprescribing (87%), the intake and use of medications (83%), desired changes to medications (78%), and the purpose of medications (74%) (Table S1). These topics were consistently rated as important by the majority of patients. The most commonly omitted topics were patient concerns about their medications (52%) and health complaints they experienced (39%). Despite not being addressed, these were also considered important by three quarters of the patients.

In the interviews with HCPs, CPs highlighted clear communication about deprescribing, emphasizing follow‐up questions to address patient concerns [Table 2, CP‐Q11]. They noted that patients' motivation facilitates deprescribing [Table 2, CP‐Q12], while asymptomatic patients pose challenges [Table 2, CP‐Q13]. Practical difficulties include managing multiple actions [Table 2, CP‐Q14], and deprescribing varied across medication classes due to differing concerns and uncertainty regarding negative outcomes [Table 2, CP‐Q15, CP‐Q16].

GPs were reluctant to adjust medications initiated by specialists [Table 2, GP‐Q17]. They valued shared responsibility with CPs [Table 2, GP‐Q18]. GPs also noted that patients did not always fully understand the rationale for stopping a particular medication [Table 2, GP‐Q19]

### 3.4 Practicality

On average, the initial patient consultation by the CP lasted 50 min (IQR 30–75) and pharmacotherapeutic analysis 35 min (IQR 5–120), discussions with GPs (including the development of a pharmaceutical care plan) 20 min (IQR 5–60) and the follow‐up 12 min (IQR 1–30). In total, the intervention required just under 120 min on average per patient (IQR 41–315) (outcome 4.1). The initial consultation took place at the patient's home in 71% of cases (outcome 4.2). For patients not seen at home, consultations were conducted at the pharmacy. The location was determined based on patient preference and feasibility.

In interviews, HCPs highlighted time and resource‐related challenges (outcome 4.3) when asked about the extent to which the intervention could be delivered given situational constraints. CPs found one CMR per week feasible; higher frequency strained capacity [Table 2, CP‐Q20]. They emphasized the role of pharmacy technicians in patient invitations and coordination with GPs because delays in GP responses were time‐consuming and demotivating [Table 2, CP‐Q21].

GPs also considered time as a major constraint, noting that deprescribing discussions with CPs required significant effort [Table 2, GP‐Q22]. They valued trust in CPs [Table 2, GP‐Q23] but found responsibility for deprescribing decisions unclear.

### 3.5 Adaptation

Adaptation of the CMR to a ‘deprescribing focused’ intervention was evaluated in the HCP interviews (outcome 5.1). Both CPs and GPs viewed the intervention as valuable for promoting a critical view of medication use and emphasized that deprescribing should always be part of a CMR [Table 2, GP‐Q24]. CPs noted that the deprescribing focus streamlined patient consultations [Table 2, CP‐Q25].

### 3.6 Limited Efficacy

On average, the intervention led to 1.3 medications stopped or reduced per patient among 20 patients with complete data (outcome 6.1). Table 3 details the top 10 most frequently deprescribed medication groups.

**TABLE 3:** Top 10 most deprescribed medication groups^a^ (ATC2 level) (n = 20 patients^b^).

Medication		Deprescribing n (%)		
Group name	ATC2 code	Total	Stopped	Reduced
		26 (100)	14 (100)	12 (100)
Drug for acid‐related disorders	A02	5 (19)	2 (14)	3 (25)
Lipid modifying agents	C10	4 (15)	3 (21)	1 (8)
Mineral supplements	A12	2 (8)	0 (0)	2 (17)
Diuretics	C03	2 (8)	1 (7)	1 (8)
Beta blocking agents	C07	2 (8)	0 (0)	2 (17)
Drugs for bone diseases	M05	2 (8)	2 (14)	0 (0)
Antidiabetic agents	A10	1 (4)	1 (7)	0 (0)
Antithrombotic agents	B01	1 (4)	0 (0)	1 (8)
Cardiac therapy	C01	1 (4)	1 (7)	0 (0)
Calcium channel blockers	C08	1 (4)	0 (0)	1 (8)

^a^ Includes only medications dispensed within the MDD system, as these were assumed to be used chronically.

^b^ Data analysed for 20 of 24 included patients: 3 had no data, and 1 had incomplete data due to death.

CPs registered deprescribing recommendations for 88% of the patients (21 out of 24) (outcome 6.2). Deprescribing actions were implemented for 83% of the patients (20 out of 24) (outcome 6.3). Overall, 70% of the DRPs identified were linked to deprescribing (Table 4), with overtreatment (*n* = 40), adverse effects (*n* = 16), and incorrect dosages (n = 16) as the most common categories (outcome 6.4).

**TABLE 4:** Number and type of DRPs and deprescribing focus

DRP type	Deprescribing		Total
	*n* (%)		*n*
	Yes	No	*n* = 99
Overtreatment	40 (100)	0 (0)	40
(potential) adverse effect	13 (81)	3 (19)	16
Incorrect dosage	12 (75)	4 (25)	16
Undertreatment	0 (0)	13 (100)	13
Inconvenience of use	2 (25)	6 (75)	8
Drug not effective	2 (40)	3 (60)	5
Contra indication or interaction	0 (0)	1 (100)	1
**Total**	**69 (70)**	**30 (30)**	**99**

The total implementation rate across all actions was 72%, with a slightly higher implementation rate of 74% specifically in deprescribing actions, as shown in Table S2 (outcome 6.5)

A total of 21 patients completed the questionnaire at both baseline and after 3 months. At baseline, participants reported an average of 3.1 ± 2.0 health‐related complaints with impact on daily life (outcome 6.6). After 3 months, this slightly decreased to 2.9 ± 2.3. The most frequently reported complaints at baseline were fatigue (17%), mobility issues (14%), and pain (9%) and remained the top‐three complaints after 3 months, though the order shifted to pain (19%), mobility issues (19%), and fatigue (18%).

## 4 Discussion

This feasibility study evaluated a deprescribing‐focused CMR among older patients with hyperpolypharmacy using Bowen's framework. Overall, the intervention was well‐received by CPs, GPs, and patients. Most patients indicated that they would recommend the intervention to others. Both CPs and GPs were motivated to participate, driven by a shared commitment to address overuse in older patients. Deprescribing recommendations were made for 88% of the patients, with at least one recommendation implemented for 83% of all patients. On average, 1.3 medications per patient were either stopped or reduced within 3 months post‐intervention. Findings across the different domains of Bowen's framework indicate that the intervention was largely *acceptable* and aligned with patient and HCP *demand*. However, these results should be interpreted within the context of motivated and experienced HCPs. Moreover, further challenges remain in the domains of *acceptability, implementation*, and *practicality*, where barriers for more sustainable integration were present, particularly related to the effective conduct of consultations, organizational constraints, and clinical experience.

Within the *implementation* domain, improving the quality of consultations between patients and CPs offers an opportunity to facilitate effective discussion of deprescribing options [15]. Although training improved CPs' ability to discuss pros and cons of deprescribing, consultations often failed to fully meet patients' information needs and did not always address personal concerns. CPs found it particularly difficult to engage asymptomatic patients in deprescribing, a challenge also highlighted in previous studies [12, 15]. To bridge this gap, CPs need to further develop consultation skills that actively elicit patient concerns, clarify the rationale for deprescribing, and incorporate motivational interviewing techniques. At the same time, patients may benefit from support in expressing their priorities; research shows that when equipped with knowledge, skills, and a supportive environment, their participation and the quality of consultations improve [36]. These challenges reflect general needs in deprescribing practice that require ongoing training and experience, with our intervention providing an initial step. Strengthening these competencies on both sides is essential to foster effective consultations, shared decision‐making and ensure deprescribing is clinically appropriate and meaningful for patients.

The second main issue is related to lack of time, within the *practicality* domain. In the current context of staff shortages and medicine shortages, HCPs perceived the required time investment as substantial, even though CMR is a routine intervention in Dutch primary care—typically over 50 CMRs per pharmacy per year, each requiring 1–1.5 h [37]. Yet this time investment should be considered in light of the broader benefits of CMRs, including improved medication safety, adherence, and treatment effectiveness [19]. Nevertheless, competing demands remain a challenge, as other studies indicate that a considerable share of CPs' time is taken up by mandatory activities and tasks resulting from insufficient staffing, including dispensing [38, 39]. As a result, patient care activities are frequently regarded as secondary and must compete with dispensing duties. To better embed patient care responsibilities into routine practice, pharmacies may need to restructure workflows, for example by separating logistical tasks. Time pressures also surfaced in the *implementation* domain, where CPs struggled to align multiple deprescribing actions and ensure structured follow‐up within daily workflow, as also reported in previous deprescribing research [15]. This highlights the need for better interprofessional collaboration and explicit agreements on roles and accountability, ideally evaluated periodically within PTAMs. Clearer allocation of responsibilities may allow CPs to implement certain deprescribing actions more independently, thereby streamlining collaboration and improving overall efficiency, a need emphasized in earlier research [40].

The third barrier, situated within the *implementation* domain, concerned CPs' clinical experience. CPs reported concerns about clinical consequences of deprescribing certain medication groups, resonating with evidence that variability in knowledge, experience, and confidence influences HCPs' willingness to deprescribe [15]. This also affected the *acceptability* domain, as some GPs questioned CPs' clinical judgement and role in deprescribing. Addressing this barrier requires ongoing, practice‐oriented training and interprofessional discussions of real‐world cases (e.g., in PTAMs or peer reflection meetings) to align perspectives, strengthen collaboration, and build shared confidence in deprescribing decisions [41].

Despite the barriers identified, this study shows that a deprescribing‐focused CMR is both feasible and valuable to pursue further. The intervention showed high *acceptability* among both patients and HCPs, supported by its collaborative approach and by patients' satisfaction with the deprescribing consultation. This is particularly encouraging and highlights the need to increase awareness of the urgency and potential benefits of the intervention towards the target group that stands to benefit most from deprescribing, thereby enabling broader uptake and impact [42, 43, 44]. Regarding *potential efficacy*, through the provided training and toolbox, motivated HCPs were able to implement deprescribing effectively, resulting in a higher average number of deprescribed medications per patient (1.3) compared to studies evaluating the effects of CMRs without a specific focus on deprescribing [20]. Importantly, CMRs address more than deprescribing alone, offering opportunities to tackle a wider range of DRPs. Although the intervention required additional time, deprescribing may reduce adverse drug events and unnecessary consultations, offsetting the initial investment. Preliminary findings indicated no deterioration in health status, in line with earlier research [20], but larger controlled studies are needed to confirm long‐term benefits on medication use, health‐related complaints, and quality of life. Embedding deprescribing within the CMR proved feasible in interested HCPs, showing that the established structure of patient consultation, CP–GP collaboration, and follow‐up offers favourable conditions for *implementation*. Previous studies confirm that pharmacist‐led deprescribing can reduce inappropriate medication use, but often struggles with limited prescriber collaboration or patient engagement [10, 11, 44]. Altogether, our results suggest that integrating deprescribing within a CMR is feasible in settings with motivated and experienced HCPs, but further research is needed to evaluate its adaptability to a broader range of routine primary care settings.

### 4.1 Strengths and Limitations

A key strength of this study is its use of mixed methods, combining both quantitative and qualitative data for a comprehensive view of the intervention's feasibility. The application of Bowen's feasibility framework structured the data and results, allowing for a systematic assessment.

Several limitations must be acknowledged. First, the small sample size (five pharmacists and 24 patients) was sufficient to explore feasibility but potentially limits the generalizability of findings. In addition, the lack of detailed HCP characteristics further restricts conclusions about representativeness. Selection bias may also have occurred, as only highly motivated pharmacists and patients participated. Those who agreed to participate may have been more receptive to deprescribing, while more skeptical HCPs and patients declined participation. For the GP interviews, pharmacists selected GPs with whom they already had established collaborations, which may have resulted in more optimistic views. This may have led to an overestimation of feasibility in routine practice. The lack of a control group limits the strength of the conclusions regarding intervention efficacy, and the three‐month follow‐up period is relatively short for assessing the long‐term effects of the intervention. Moreover, the study was conducted within the Dutch healthcare system, where structured GP–CP collaboration is common. This context may limit transferability to other healthcare systems.

## 5 Conclusion

This study demonstrates that, within a setting of motivated HCPs experienced in conducting CMRs, incorporating a focus on deprescribing into a CMR for older patients with hyperpolypharmacy was feasible and well received by both patients and HCPs. Feasibility was supported by high acceptability, strong demand from both groups, and demonstrated potential for deprescribing, while barriers emerged in the domains of implementation and practicality. For deprescribing to become part of routine care for older patients, consultation skills and interprofessional collaboration need to be strengthened, alongside exploration of this approach's feasibility and sustainability across a broader range of primary care settings.

## Author Contributions

**Gert Baas:** conceptualization, methodology, investigation, formal analysis, writing – original draft preparation. **Mette Heringa:** conceptualization, methodology, investigation, formal analysis, writing – reviewing and editing. **Sanne Verdoorn:** conceptualization, methodology, investigation, formal analysis, writing – reviewing and editing. **Henk‐Frans Kwint:** conceptualization, methodology, writing – reviewing and editing. **Eman Badawy:** conceptualization, investigation, formal analysis, writing – reviewing and editing. **Jacobijn Gussekloo:** conceptualization, methodology, writing – reviewing and editing. **Marcel Bouvy:** conceptualization, methodology, writing – reviewing and editing.

## Funding

The Dutch organization ZonMw funded the study (project number 10140021910504), as part of the programme ‘Goed Gebruik Geneesmiddelen’ and has no other role. ZonMw is the Dutch national organization for health research and healthcare innovation.

## Conflicts of Interest

The authors declare no conflicts of interest.

## Supporting information


**Data S1:** PREM questionnaire

**Data S2:** Topic guides interviews

**Data S3:** Training evaluation questionnaire

**Table S1:** Overview of topics (not) discussed during patient consultation

**Table S2:** Type of actions recommended and implemented, subdivided by whether this concerned deprescribing or not

## Data Availability

The data that support the findings of this study are available from the corresponding author upon reasonable request.
