# Supplementary material for: A Clinical Medication Review Focused on Deprescribing in Older Patients With Hyperpolypharmacy: A Mixed‐Methods Feasibility Study
Source: Basic Clin Pharmacol Toxicol. 2025 Dec 28;138(2):e70184. doi: 10.1111/bcpt.70184 (PMC12744689; doi:10.1111/bcpt.70184)
Supplement: Supplementary file 1 — Data S1: PREM questionnaire [file BCPT-138-0-s004.docx]

**SUPPLEMENTARY INFORMATION 1.** PREM QUESTIONNAIRE

You have had one or more conversations with the pharmacist about your medication, during which you may have received advice or adjustments to your medication use. We would like to ask you a few questions regarding these conversations with the pharmacist.

1. **To what extent do you agree with the following statements?**

|  | **Totally disagree** | **Disagree** | **Neutral** | **Agree** | **Totally agree** | **Not applicable** |
| --- | --- | --- | --- | --- | --- | --- |
| The pharmacist understood my questions about my health and medications. |  |  |  |  |  |  |
| The information the pharmacist provided about reducing and stopping medication was difficult to understand |  |  |  |  |  |  |
| The pharmacist took my wishes into account when adjusting the medications. |  |  |  |  |  |  |
| I got advice from the pharmacist on what I can do something with. |  |  |  |  |  |  |
| I thought the conversation with the pharmacist was unnecessary. |  |  |  |  |  |  |
| I have confidence in the pharmacist. |  |  |  |  |  |  |

1. **How important did you find the following topics during your conversation with the pharmacist?**

*(if not discussed, please select two options: importance & topic not discussed)*

|  | **Not important at all** | **Not important** | **Neutral** | **Important** | **Very important** | **Topic not discussed** |
| --- | --- | --- | --- | --- | --- | --- |
| What I would like to change about my medications. |  |  |  |  |  |  |
| The pros and cons of reducing and/or stopping medication |  |  |  |  |  |  |
| What the use of my medications is |  |  |  |  |  |  |
| The concerns I have about my medications |  |  |  |  |  |  |
| The questions I have about the medications |  |  |  |  |  |  |
| My satisfaction with the medications |  |  |  |  |  |  |
| The intake/use of the medications |  |  |  |  |  |  |
| What health problems do I suffer from |  |  |  |  |  |  |
| Which health problems may be side effects of my medication |  |  |  |  |  |  |

1. **General assessment of the conversation with the pharmacist**
2. Would you recommend the conversation with the pharmacist to other people? on a scale of 0 to 10, where 0 means you would definitely not recommend it, and 10 means you would definitely recommend it.


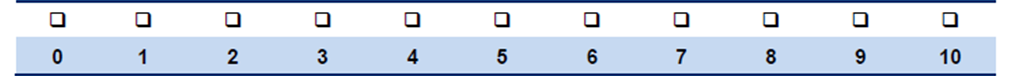


1. Do you have any positive comments or points for improvement (feedback) regarding the conversation with the pharmacist?
