# Supplementary material for: A Clinical Medication Review Focused on Deprescribing in Older Patients With Hyperpolypharmacy: A Mixed‐Methods Feasibility Study
Source: Basic Clin Pharmacol Toxicol. 2025 Dec 28;138(2):e70184. doi: 10.1111/bcpt.70184 (PMC12744689; doi:10.1111/bcpt.70184)
Supplement: Supplementary file 2 — Data S2: Topic guides interviews [file BCPT-138-0-s005.docx]

**SUPPLEMENTARY INFORMATION 2:** TOPIC GUIDES INTERVIEWS

**TABLE A:** Patient interview

| **Reasons for participation** |
| --- |
| What was the reason you decided to participate in the study? |
| How did you feel about the pharmacist calling you to participate in this study? |
| **Information and expectations** |
| The pharmacists also sent you information about the study by post. Did you read it? If so, what did you think? If not, what was the reason you didn't read it?  If the patient does not know what information you are talking about, name the following documents point by point: invitation letter, information flyer, research in steps and information letter. |
| Could you explain in your own words what was expected of you? If so, what was exactly clear? If not, what wasn't clear? And why? |
| **Experience with the deprescribing consult** |
| During the conversation with your pharmacist, you discussed reducing and stopping your medication. How do you feel about having a conversation about reducing and stopping medication? Do you think it's useful? (Why or why not?) |
| Have any adjustments been made to your medication after the consultation? If so, did you feel that you were able to participate in the decision-making process regarding these changes?  What did you think of the way in which these adjustments were implemented? Did the pharmacist provide you with sufficient information? ( For example, about the advantages and disadvantages?) If not, do you know why? |
| Would you like to have another conversation about your medication in the future to see if the medications can be stopped or reduced? Why or why not? Would you recommend such a conversation to others? |
| **Experience with questionnaires and participation** |
| You have now received and answered 3 questionnaires . How did you experience filling this in?  What did you think of the questions you were asked? |
| What did you think of the questionnaires being conducted by telephone? |
| Did you ever doubt whether you wanted to continue during the investigation? If so, what caused those doubts? What made you go ahead with the research? |
| Which aspects of the study were positive? Why do you think that?  Which aspects of the study were negative? Why do you think that?  Do you have any suggestions for improvement/tips for the researchers or the pharmacist? |

**TABLE B:** Community pharmacist interview

| **Experience with training** |
| --- |
| At the beginning of the study, you attended a training on reducing and stopping medications, communication, and organization. How did you experience this day?   - 1. During the implementation of the medication review, were there any specific areas where you felt you lacked knowledge or skills? Could the training have helped you in these areas?   2. Were there other topics (related to knowledge or skills) that you think would have been beneficial to include in the training?" |
| The training covered the technical aspects of the research as well. Was it clear what we expect from you as a participating pharmacist? What were the points that could be made even clearer? |
| **Use of toolbox materials** |
| After the training, you received the Pharmaceutical Therapeutic Audit Meetings (PTAM) presentation.  Have you used this presentation? What did you think of this? |
| Did you use the knowledge documents and risk assessment tools during the medication reviews?  If not, why not? If so, which ones? And what did you think of this? |
| Did you use the deprescribing conversation protocol?  If not, why not? If so, what did you think of this? |
| Was the research protocol clear? If not, what do you think is missing? |
| **Patient selection and recruitment** |
| Can you tell us how the patient selection process went?  What were reasons for not inviting someone? Were there enough patients to invite? How did the collaboration with the GP go? How were control patients selected? |
| How did you invite the patients?  Approximately how many people did not want to participate? Have you also registered this in the selection table? (if not, please do)  What were the reasons patients gave for not wanting to participate? |
| What did you think of the selection criteria? |
| How would you adjust the criteria to reach a relevant audience? |
| **Conducting deprescribing conversations** |
| How did the conversation with the patient go, where the focus was on reducing and stopping medication? Did this change as you had multiple conversations? |
| During the training, attention was also paid to the patient's wishes/goals and the prioritization of FTPs.  How did the discussion of the patient's wishes go? |
| And how did you ensure that the patient could participate in the decision-making process? |
| Did the conversations deviate from what you normally do in medication reviews? |
| **Conduct of CMR focused on deprescribing** |
| Would you like to continue to conduct medication reviews with a focus on reducing and stopping medication? Why or why not? |
| Do you think the intervention (i.e. conducting a medication review with a focus on reducing and stopping medications) is feasible? |
| Would you recommend it to colleagues? If so, why? If not, what would need to be changed? |
| How did the collaboration with the GP/ practice nurse go? |
| How was the feedback and internal communication organised? |
| Was this different from "normal" medication reviews? |
| Have your proposals for reducing and stopping been adopted? Why or why not? |
| **Knowledge, skills and clinical reasoning** |
| Did you have sufficient knowledge/tools to reduce/stop medication? If not, what else were you missing? |
| Have you used the medication roll while stopping/tapering off medications? If so, how did you do it? If not, why has it not been used? |
| How did the follow-up interview with the patient go?  Were there any tasks divided between you and the GP ? How was communication organised between you and the GP? |
| **Evaluation** |
| Which aspects of the study were positive? Why?  Which aspects of the study were negative? Why? |

**TABLE C:** General practitioner interview

| **Overall perspective and attitudes towards deprescribing** |
| --- |
| What did you think of the PTAM on deprescribing? |
| How useful do you find this intervention? (a medication review focused on deprescribing) |
| How do you view “deprescribing”? What do you think are hindering or promoting factors in carrying this out? |
| **Patient selection** |
| What did you think of the selection criteria (>75 years, medication roll and more than 10 medications)? |
| How would you adjust the criteria to reach a relevant audience? |
| **Use of toolbox materials** |
| Have you also used the knowledge documents on the stopping/phasing out of medicines?  If so, what did you get out of these documents? If not, why not? |
| How did the collaboration with the pharmacist go in terms of patient selection? |
| How was the feedback and internal communication organised? Was this different from "normal" medication reviews? |
| What did you think of the proposals on stopping/tapering off medicines? Why or why not? |
| **Feasibility of implementation in daily practice** |
| To what extent did you find the intervention feasible? (e.g. selecting patients, consulting with the pharmacist and registration/monitoring in GP-information system). |
| 1. Which aspects of the study were positive? Why? 2. What aspects of the study did you find negative? Why? |
