# Supplementary material for: A Clinical Medication Review Focused on Deprescribing in Older Patients With Hyperpolypharmacy: A Mixed‐Methods Feasibility Study
Source: Basic Clin Pharmacol Toxicol. 2025 Dec 28;138(2):e70184. doi: 10.1111/bcpt.70184 (PMC12744689; doi:10.1111/bcpt.70184)
Supplement: Supplementary file 3 — Data S3: Training evaluation questionnaire [file BCPT-138-0-s002.docx]

**SUPPLEMENTARY INFORMATION 3.** TRAINING EVALUATION QUESTIONNAIRE

1. Which part of the training did you find most useful, and why?
2. Which part of the training did you find the least useful, and why?
3. Please indicate below to what extent you agree with the following statements:

|  | **Totally disagree** | **Disagree** | **Neutral** | **Agree** | **Totally agree** |
| --- | --- | --- | --- | --- | --- |
|  | **N** | | | | |
| 1. **After following the training module on *organization...*** |  | | | | |
| … I am able to carry out the intervention in the context of the practical research in accordance with the protocol. | 0 | 0 | 0 | 6 | 0 |
| …I am informed about the research design. | 0 | 0 | 0 | 3 | 3 |
| … I can organize a medication review focused on deprescribing, including follow-up, and effectively collaborate with the general practitioner and practice nurse. | 0 | 0 | 0 | 4 | 2 |
| 1. **After following the training module on *consulting...*** |  | | | | |
| … I am able to introduce the topic of deprescribing in a conversation with the patient. | 0 | 0 | 0 | 5 | 1 |
| …I am able to gain insight into the wishes and perceptions of the patient regarding deprescribing. | 0 | 0 | 0 | 4 | 2 |
| 1. **After following the training module on *deprescribing...*** |  | | | | |
| … I am able to apply the information from the knowledge documents in practice when implementing and monitoring deprescribing for older adults. | 0 | 0 | 2 | 3 | 1 |
| …I can develop a pharmacotherapeutic treatment plan, including prioritization and monitoring of actions, during a medication review focused on deprescribing | 0 | 0 | 1 | 3 | 2 |
| 1. **The training met my expectations** | 0 | 0 | 1 | 3 | 2 |

4. What areas do you think could be improved?

**Thank you for taking the time to provide your feedback!**
