# Supplementary material for: A Clinical Medication Review Focused on Deprescribing in Older Patients With Hyperpolypharmacy: A Mixed‐Methods Feasibility Study
Source: Basic Clin Pharmacol Toxicol. 2025 Dec 28;138(2):e70184. doi: 10.1111/bcpt.70184 (PMC12744689; doi:10.1111/bcpt.70184)
Supplement: Supplementary file 4 — Table S1: Overview of topics (not) discussed during patient consultation [file BCPT-138-0-s001.docx]

**SUPPLEMENTARY TABLE S1.** Overview of topics (not) discussed during patient consultation

| **Statement** | **Discussed**  **N (%)** | | | | **Not discussed**  **N (%)** | | | |
| --- | --- | --- | --- | --- | --- | --- | --- | --- |
|  | **Not important  (at all)** | **Neutral** | **(Very) important** | **Unknown** | **Not important  (at all)** | **Neutral** | **(Very) important** | **Unknown** |
| The (dis)advantages of deprescribing | 20 (87) | | | | 3 (13) | | | |
|  | 0 (0) | 2 (10) | 18 (90) | 0 (0) | 1 (33) | 0 | 1 (33) | 1 (33) |
| Intake/use of medications | 19 (83) | | | | 4 (17) | | | |
|  | 1 (5) | 5 (26) | 13 (68) | 0 (0) | 3 (75) | 0 (0) | 1 (25) | 0 (0) |
| What I would like to change about my medications | 18 (78) | | | | 5 (22) | | | |
|  | 1 (6) | 4 (22) | 13 (72) | 0 (0) | 1 (20) | 0 (0) | 3 (60) | 1 (20) |
| Purpose of my medications | 17 (74) | | | | 6 (26) | | | |
|  | 1 (6) | 2 (12) | 14 (82) | 0 (0) | 2 (33) | 0 (0) | 3 (50) | 1 (17) |
| Questions I have about the medications | 16 (70) | | | | 7 (30) | | | |
|  | 0 (0) | 2 (13) | 14 (88) | 0 (0) | 1 (14) | 0 (0) | 4 (57) | 2 (29) |
| Satisfaction with the medications | 16 (70) | | | | 7 (30) | | | |
|  | 0 (0) | 7 (44) | 9 (56) | 0 (0) | 2 (29) | 1 (14) | 3 (43) | 1 (14) |
| Which health complaints could be side effects of my medications | 15 (65) | | | | 8 (35) | | | |
|  | 1 (7) | 1 (7) | 13 (87) | 0 (0) | 1 (13) | 0 (0) | 6 (75) | 1 (13) |
| Which health complaints I am experiencing | 14 (61) | | | | 9 (39) | | | |
|  | 2 (14) | 1 (7) | 11 (79) | 0 (0) | 5 (56) | 1 (11) | 2 (22) | 1 (11) |
| Concerns I have about my medications | 11 (48) | | | | 12 (52) | | | |
|  | 1 (9) | 4 (36) | 6 (55) | 0 (0) | 5 (42) | 1 (8) | 4 (33) | 2 (17) |
