# Supplementary material for: A Clinical Medication Review Focused on Deprescribing in Older Patients With Hyperpolypharmacy: A Mixed‐Methods Feasibility Study
Source: Basic Clin Pharmacol Toxicol. 2025 Dec 28;138(2):e70184. doi: 10.1111/bcpt.70184 (PMC12744689; doi:10.1111/bcpt.70184)
Supplement: Supplementary file 5 — Table S2: Type of actions recommended and implemented, subdivided by whether this concerned deprescribing or not [file BCPT-138-0-s003.docx]

**SUPPLEMENTARY TABLE S2.** Type of actions recommended and implemented, subdivided by whether this concerned deprescribing or not

| **Type of action** | **Total** | | | **Deprescribing** | | |
| --- | --- | --- | --- | --- | --- | --- |
|  | **Recommended**  **N** | **Implemented**  **N** | **Implementation rate (%)** | **Recommended**  **N** | **Implemented**  **N** | **Implementation rate (%)** |
| Stop medication | 46 | 33 | 72 | 46 | 33 | 72 |
| Decrease dosage | 21 | 15 | 71 | 20 | 15 | 75 |
| Start medication | 8 | 6 | 75 | - | - |  |
| Substitute medication | 6 | 5 | 83 | - | - |  |
| Other | 5 | 4 | 80 | - | - |  |
| Provide advice/information | 4 | 2 | 50 | 1 | 1 | 100 |
| Referral to another healthcare provider | 4 | 4 | 100 | 2 | 2 | 100 |
| Increase dosage | 2 | 1 | 50 | - | - |  |
| Assessing medication adherence and usage | 2 | 0 | 0 | - | - |  |
| Request clinical measurement | 1 | 1 | 100 | - | - |  |
| **Total** | **99** | **71** | **72** | **69** | **51** | **74** |
